# Cutaneous T-Cell Lymphoma (CTCL) Arising Post Kidney Transplant: A Review of Clinical Variants in the Literature

**DOI:** 10.3390/hematolrep16010002

**Published:** 2023-12-28

**Authors:** Jordan Pilkington, Sara Shalin, Henry K. Wong

**Affiliations:** 1 College of Medicine, University of Arkansas for Medical Sciences: 1, Little Rock, AR 72205, USA; jtpilkington@uams.edu; 2 Department of Dermatology, University of Arkansas for Medical Sciences, Little Rock, AR 72205, USA; scshalin@uams.edu; 3 Central Arkansas Veterans Health System, Little Rock, AR 72205, USA

**Keywords:** post-transplant lymphoproliferative disease, transplant, cutaneous T-cell lymphoma, mycosis fungoides, kidney transplant

## Abstract

Post-transplant lymphoproliferative disease is a rare disorder with an annual incidence of 0.5% to 3.7%. Development of this disorder carries with it a poor prognosis. In this report, we describe a rare case of post-transplant primary cutaneous T-cell lymphoma (PT-CTCL) mycosis fungoides stage IIB in a patient following kidney transplantation, as well as a review of PT-CTCL reported in the literature. The treatment following diagnosis included bexarotene, cyclosporine, and prednisone. Currently, the patient is free from disease. This information aims to add to the knowledge of the prevalence and management of PT-CTCL.

## 1. Introduction

The success of solid organ transplantation is supported by a continuing progressive increase each year. In 2022, the Organ Procurement and Transplant Network (OPTN) reported 42,888 transplant procedures performed, with 25,499 of those consisting of kidney transplants. The mortality rate for first-time recipients of a kidney transplant is 20.5%, with the risk declining as the time since transplant increases. Cardiovascular disease is the main cause of death in kidney transplants, with cancer-related death comprising the second most common cause [1]. A rare complication from transplantation includes the development of post-transplant lymphoproliferative disease (PTLD), which has an annual incidence of 0.5% to 3.7% [2]. The incidence of PTLD varies and is dependent on the organ transplanted, with multiorgan/intestinal transplants being most frequent and kidney transplant being the least frequent [3].

PTLDs have been grouped by the World Health Organization into four different subtypes, including early lesions, polymorphic PTLD, monomorphic PTLD, and classic Hodgkin lymphoma-type PTLD. PTLD originating from T-cell lineage is less common. Additionally, primary cutaneous PTLDs of both B- and T-cell origins comprise the least frequent subgroup of PTLDs, with fewer than 100 reports found in the literature [4].

This report describes a rare case of post-transplant primary cutaneous T-cell lymphoma (PT-CTCL) of mycosis fungoides stage IIB development following kidney transplantation. Information regarding this subject contributes to the knowledge of treatments for patients with this condition.

## 2. Case Report

**Figure 1:**
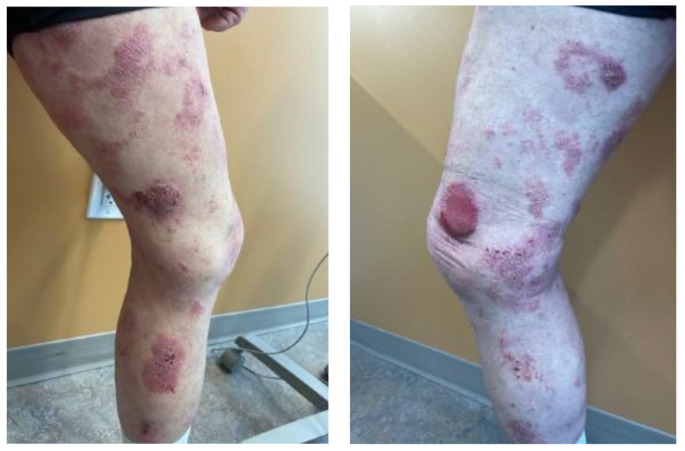
Skin manifestation. Extensive involvement of CTCL with tumor, plaques and patches affecting bilateral lower legs. Thick plaques and patches (left panel) shown on the right leg. Thick eroded tumor present on the right anteromedial knee (right panel) showed maximal uptake on the PET-CT scan (Figure 3).

**Figure 2:**
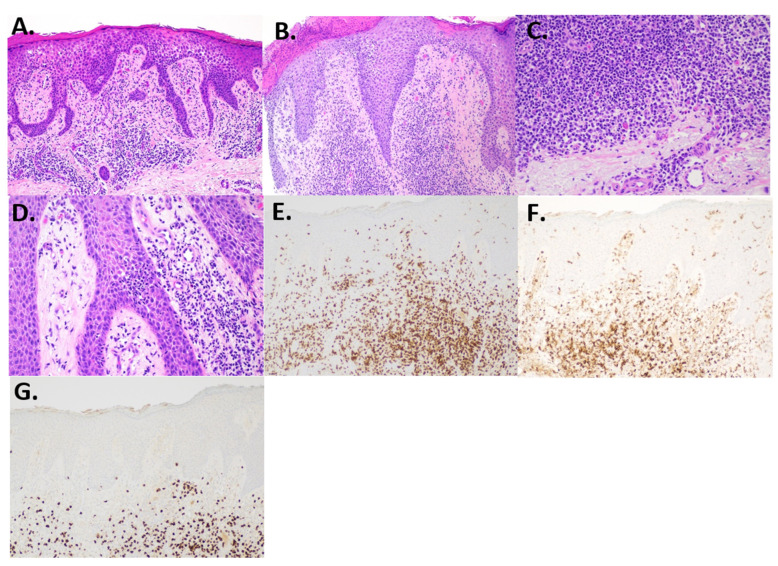
Histology and immunohistochemistry. (**A**) Initial biopsy showing epidermal hyperplasia, spongiosis, and a mixed dermal inflammatory infiltrate (H and E, 100×). (**B**) Second biopsy showed acanthosis and spongiosis with some lymphocyte exocytosis (H and E, 100×). (**C**) Dermal infiltrate showed small lymphocytes, many plasma cells, and rare eosinophils (H and E, 200×). (**D**) Focal epidermotropism with slight lymphocyte atypia (H and E, 200×). (**E**) CD3-highlighted T-cells, including some within the epidermis (CD3, 100×). (**F**) CD4 shows predominance over CD8. (**G**) CD8 is reduced compared to CD4 (100×).

**Figure 3:**
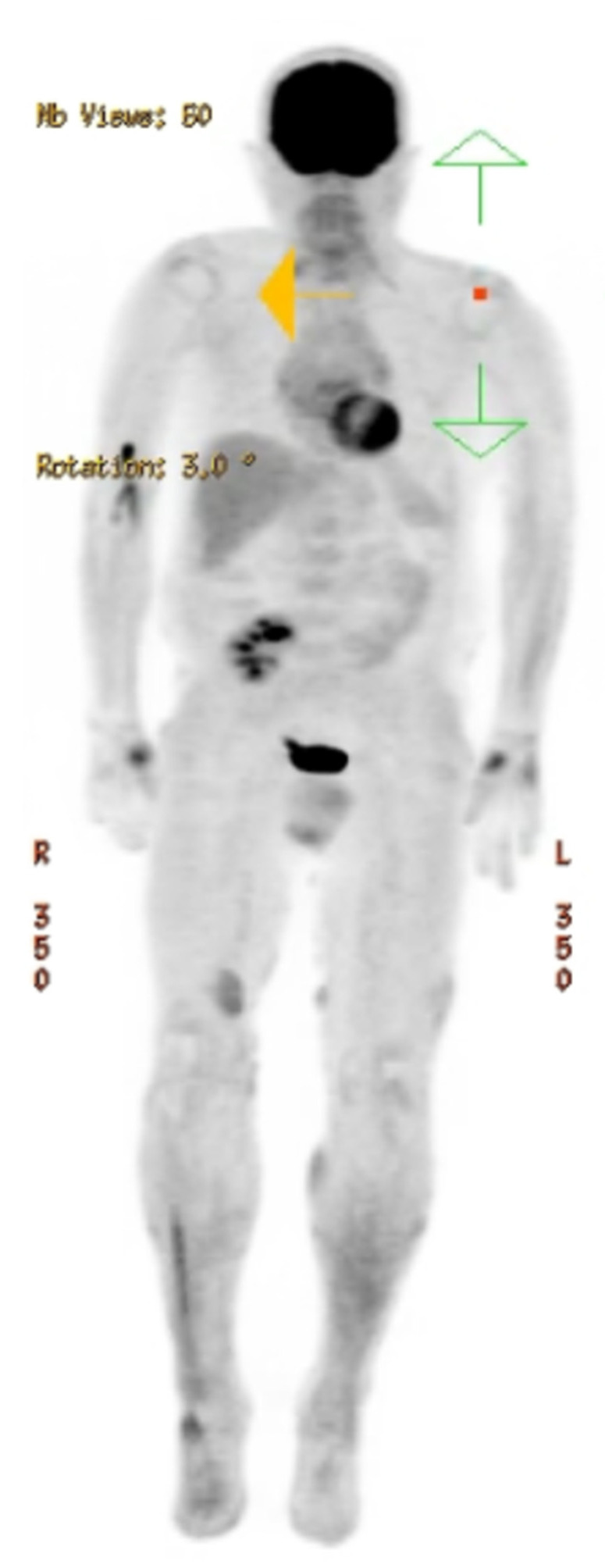
Radiologic imaging. PET-CT vertex to feet showing multiple areas of cutaneous skin increased SUV signal enhancement in the bilateral lower extremities, particularly in the right distal anteromedial leg. No evidence of avid lymphadenopathy or abnormal signal enhancement in the spleen.

A 61-year-old male with a history of kidney transplantation in October of 2012 from stage two hypertension-induced end stage renal disease (ESRD) presented to the dermatology outpatient clinic with a concern of a greater than 2-year rash and tumor of the right leg. At presentation, the patient was 9 years post-transplant and his immunosuppression regimen included mycophenolate mofetil 250 mg twice daily, cyclosporine 100 mg twice daily, and prednisone 5 mg daily. Clinical examination showed numerous red to violaceous indurated plaques scattered on the flanks and proximal upper and lower extremities with scale and secondary impetiginization (Figure 1). Prominently, a large 5+ cm tumor with drainage was present on the right medial thigh. Prior to presentation, the patient had been self-treating the rash with over-the-counter (OTC) hydrocortisone that initially controlled the spread and itch. Initial biopsy of the rash showed robust spongiotic dermatitis with minimal epidermotropism and a mixed dermal inflammatory infiltrate, favored to represent an eczematous drug eruption (Figure 2A). Given the persistent clinical concern for CTCL, additional biopsies and immunophenotyping were performed a year later, which again showed similar histopathology with marked epidermal hyperplasia, prominent spongiosis, and serum crusting. The pathology also revealed focal T-lymphocytes in the epidermis, with CD4 subsets slightly predominating over CD8 (Figure 2F,G). CD30 was not detected in the neoplastic lymphocytes, and EBV in situ hybridization, performed due to the patient’s post-transplant status, was negative. A subsequent T-Cell receptor gene rearrangement assay was positive for clonality, supporting a diagnosis of spongiotic mycosis fungoides. Clinical staging to determine the presence of nodal or other organ involvement was assessed via PET-CT. The PET-CT results showed multiple areas of uptake along areas of skin, thickening on the bilateral lower extremities with maximal uptake in the right distal anteromedial leg, but did not identify nodal or visceral involvement (Figure 3). The tumor burden was consistent with ISCL stage IIB with tumor involvement (T3), a BSA of approximately 20%, and lack of involvement of nodes or other organs. These findings are consistent with primary cutaneous T-cell lymphoma without indication of metastasis.

**Figure 4:**
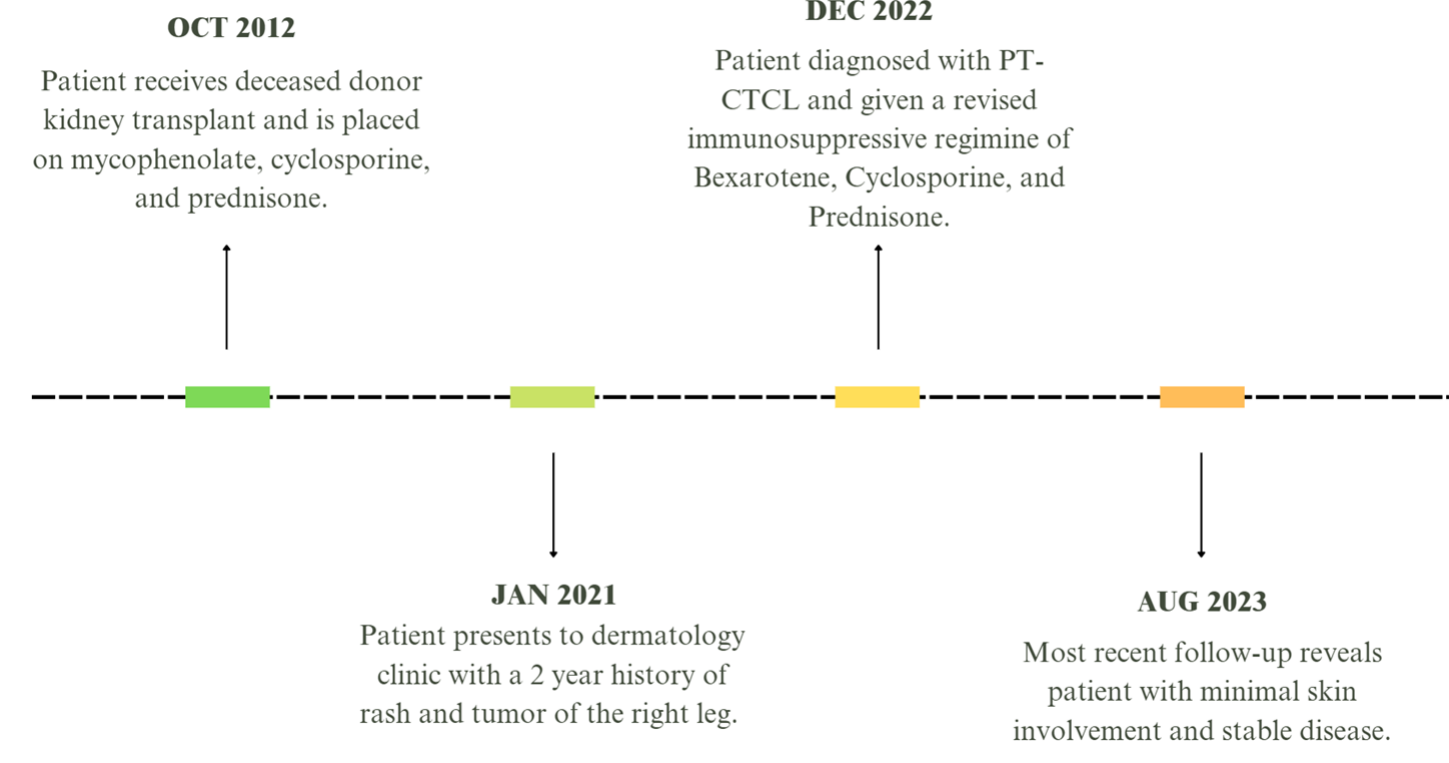
Timeline of patient presentation and treatment.

After diagnosis, the immunosuppressive regimen was revised. To treat his CTCL, the patient started on bexarotene 75 mg twice per day by mouth. Bexarotene was chosen due to its lack of immunosuppressive effects, FDA approval for prevention of CTCL progression, and reports of effectiveness in patients with PT-CTCL [5]. Cyclosporine 100 mg twice daily and prednisone 5 mg daily were continued to prevent graft rejection. Mycophenolate was discontinued. Additionally, topical corticosteroids (triamcinolone 0.1% ointment and fluocinonide 0.1% cream) were added for relief of pruritus. At the patients most recent follow-up, three years post diagnosis, the patient maintains good control of all skin lesions and has a well-controlled disease. Figure 4 can be used to best understand the overall timeline of clinical events.

## 3. Discussion

The risk of PTLD increases from altered immunity secondary to iatrogenically induced immune suppression associated with reduced tumor immune surveillance, which is necessary to the survival of the transplanted graft. Additionally, excessive immunosuppression increases the risk of serious infections. Thus, management of PTLDs is challenging due to the requirement of exquisitely balanced therapies that minimize the risk of graft rejection with undesired excessive lymphoproliferation. Some success has been achieved through a combination of reduction or alteration of immunosuppressive medication and local radiotherapy for improving graft survival, but the potential for rapidly progressing disease remains high [6].

The clinical presentation of PTLD is highly heterogenous and is influenced by immunosuppression regimens and Epstein–Barr virus (EBV) status, which together affect the overall prognosis [6,7,8,9]. Those arising from B-cell-origin lymphomas comprise the majority of PTLDs. Of those from B-cells, EBV associations can be detected in 33–48%, which have poorer prognoses [10]. EBV-associated PTLD is also linked to an early onset of disease. EBV-positive patients often develop PTLD one year post transplant, while EBV-negative patients who develop PTLD often display a late onset, between 5 and 15 years post transplant [11].

PTLD is an unfortunate complication of organ transplantation. The most common variant of PTLD originates from B-cells and develops most often early after transplant compared to PTLD originating from other immune cell types, and is often associated with EBV. In patients who develop PTLD after a prolonged post-transplant period, EBV is less frequently detected [12]. The expression of CD20 in B-cell PTLDs presents the ability for the use of targeted treatment with rituximab.

PTLDs arising from T-cells are less frequently observed. The spectrum of PTLD T-cell malignancies is heterogenous, and those that have been described are summarized in Table 1. Anaplastic large cell lymphoma (ALCL) is the most frequent variant reported in the literature (Table 1). Other less common variants include peripheral T-cell lymphoma (PTCL), which has a similar reported incidence as CTCL, as well as hepatosplenic gamma delta and rare T-cell variants (Table 1). The treatment of T-cell PTLD is challenging and the survival outcome, as summarized in Table 1, is dire for most patients.

PT-CTCL remains an infrequent malignancy, representing approximately 5% of PTLD cases involving the skin. Astute recognition of this variant is essential to comprehensively characterize the spectrum of PTLD and develop approaches for treatment. A meta-analysis describing the only 14 cases of PT-CTCL reported in the literature from 1992 to 2005 discovered the mean age of diagnosis to be 57.2 years old, with a mean time to diagnosis post transplant of 5.8 years. Of these 14 patients, 13/14 had received a renal transplant, with 1 patient receiving a heart transplant. Overall, the mortality rate was poor with six of the patients dying in less than a year and only four patients achieving complete remission. This same study looked specifically at skin findings related to MF and saw an increasing prevalence of erythroderma in the PT-CTCL patients. Frequent presentation with erythroderma and a high rate of mortality in PT-CTCL patients is uncharacteristic of MF, which more commonly includes patches, plaques, and an indolent disease course [2].

This case represents a patient that developed PT-CTCL post kidney transplant that follows an uncommon clinical progression compared to those reported in the current literature. The development of symptoms was first noted in this patient 9 years post transplant, while the final diagnosis was not determined until 10 years post transplant. This time frame is greater than 3 years past the average diagnosis of other reports from patients with the same condition. Additionally, this patient presented with patches and plaques involving 20% BSA, which contrasts previously reported cases of predominant erythroderma. According to the patient, pruritis and skin discomfort was mild and was maintained with OTC topical corticosteroids for at least a year. The clinical presentation overall was indolent with a relatively slow development of new lesions. Indolent presentation of PT-CTCL is uncommon, with reports by Ravat et al. [6] noting an average survival rate of only 10.2 months in a review of 23 patients.

In their series of eight patients from 1998 to 2013, Shimshak et.al. reported that the most common variant of PT-CTCL displays the folliculotropic variant, with positivity in CD3, CD4, and CD5, loss of CD7 expression, and negativity for CD30 and EBV [13]. The majority of patients in this study comprised liver transplant patients, which may explain the difference in presentation from previous reports of patients with erythroderma. A case series of 23 patients with PT-CTCL from Ravat et.al. reported the most common histological subtype as primary cutaneous anaplastic large cell lymphoma (PCALCL) [6]. This case series consisted of a majority of kidney transplant patients. Overall, cases of PT-CTCL exhibit a variety of clinical presentations and subtypes that may favor a specific organ transplant. A review of our case via biopsy revealed the histology of spongiotic MF in a renal transplant patient that presented with an indolent disease course. Immunohistochemistry was positive for CD3 and CD4, and negative for CD30 with negative CD7 staining.

Besides reduction of immunosuppression, no established treatment recommendations from the NCCN exist for CD30- cases of PTLD of T-cell origin. However, brentuximab with cyclophosphamide, doxorubicin, and prednisone has been suggested for CD30+ cases [14]. Due to this, the reduction, modification, or discontinuation of immunosuppressive agents is often the initial treatment approach for PT-CTCL. In addition to these therapeutic adjustments, patients with classical forms of MF are often treated with skin-directed therapies (topical corticosteroids, PUVA) [4]. Our patient’s treatment regimen consisted of reduction of immunosuppressors (cyclosporine, prednisone), discontinuation of mycophenolate, addition of bexarotene, and topical fluocinonide and triamcinolone. Bexarotene treatment was chosen as it is not immunosuppressing, is FDA-approved for CTCL to control the progression of the disease, and has been reported to be effective in patients with PT-CTCL [5]. In evaluations of our patient, PET-CT examination did not uncover systemic involvement and showed the PT-CTCL to be limited to the skin. Follow-up evaluation showed improvement in the skin lesions with bexarotene and topical treatment.

Although rare, PT-CTCL may develop during long-term treatment of transplant patients. Cutaneous lesions and rashes may be the initial presentation of this disease and can often lead to serious complications in patients, as reported among others in the literature. Skin biopsy analysis with immunohistochemistry is critical for accurate diagnoses. In this case, we presented a patient who developed spongiotic MF stage IIB nine years post kidney transplant with an indolent course, skin-limited disease and response to bexarotene and topical treatment. The management and treatment of PT-CTCL can vary, and this report contributes to the heterogenous presentation and understanding of these diseases to improve patient outcomes (Table 1).

**Table 1:** Literature review of PT-CTCL.

Author	Lymphoma Subtype	Organ Transplant	Onset Post-Transplant	Survival	Clinical Presentation	Management
Singavi [3]	CTCL mycosis fungoides type	Allogenic HSCT	6 years	1 year after Dx	Asymptomatic eczema-like cutaneous lesions	No change in therapy
Santos-Briz [15]	CTCL mycosis fungoides type	Renal	Preceded transplant	Indolent 6 years post transplant	Nodular skin lesion	Narrow band UVB (nbUVB) phototherapy
Griffin [16]	Folliculotropic mycosis fungoides	Cardiac	N/A	N/A	Inflamed and pruritic skin lesions	Oral bexarotene, nbUVB, topical retinoid
Rogers [17]	Mycosis fungoides	Small bowel and liver	10 years	DOD 1 year	Pruritic scaly plaque	Topical steroids, UVB, EPOCH chemotherapy ****
Lewis [5]	MF stage IB	Liver	5 years	Complete remission at 16 years	Annular skin lesion	Topical nitrogen mustard, bexarotene, fenofibrate, levothyroxine, ROI
Al Airoush [18]	Sezary syndrome	Liver	13 years	Not mentioned	Pruritic eczematous dermatitis	ROI
Rajakariar [19]	ALCL, ALK(-)	Renal	10 years	DOD 6 weeks	Pyrexia of unknown origin (POU) and nodular skin lesion	Methylprednisolone
Coyne [20]	ALCL, ALK (-)	Renal	2 years	DOD 18 months	Nodular skin lesions, fever	ROI; increased prednisolone and acyclovir
Coyne [20]	ALCL, ALK (-)	Renal	6 years	DOD at 15 months	Ulcerating skin lesions	VAPEC-B chemotherapy followed by radiotherapy **
Coyne [20]	ALCL, ALK (+)	Renal	9 years	DOD “several months post Dx”	Multinodular skin lesion	Discontinuation of cyclosporin; CHOP chemotherapy
Lucioni [21]	ALCL, ALK (-)	Cardiac	9 years	Complete remission at 49 mo f/u	Nodular skin lesions	External radiotherapy; ROI; IgG; IFN-alpha; CHOP
Magro [22]	ALCL, ALK(?)	Liver	15 years	DOD shortly after Dx	Lower extremity edema	Death before change of Tx
Magro [22]	ALCL, ALK(-)	Liver	7 years	Remission at 3-year f/u	Nodular skin lesions	ROI
Balachandran [23]	ALCL, ALK(-)	Renal	10 years	Complete remission at 2-year f/u	Posterior mediastinum	CHOP
Treaba [24]	ALCL, ALK(-)	Renal	6 years	Alive at 5 mo f/u with residual disease	Nausea, fever, diarrhea, vomiting, LE pitting edema	CHOP
Salama [25]	ALCL, ALK(-)	Renal	2 years	Death 22 months after Dx	Nodular skin lesions	ROI; radiation; chlorambucil
Salama [26]	ALCL, ALK(-)	Renal	8 years	DOD at 3 years	Skin nodules	ROI, herbal remedies, local radiation, etoposide
Belloni-Fortina [27]	ALCL, CD30+ ALK(ND)	Cardiac	11 years	Complete remission at 2 years	Nodular skin lesion	Surgical removal; radiotherapy
Ward [28]	ALCL, CD30+	Renal	56 months	DOD	Erythema with papules and hyperpigmented nodules	Prednisone, cyclosporin A, azathioprine
Seckin D [29]	ALCL, CD30+	Renal	10 months	Remission at 5 months	Nodule	Prednisone, cyclosporin, azathioprine
Cooper SM [30]	ALCL, CD30+	Renal	66 months	DOD	Nodules	Mycophenolate mofetil, prednisone, cyclosporin A
Kim HK [31]	ALCL, CD30+	Renal	16 years	Complete remission at 10 months	Polypoid mass	Prednisone, cyclosporin A, azathioprine
De Nisi MC [32]	ALCL, CD30+	Cardiac	60 months	Complete remission	Nodule	Mycophenolate mofetil, tacrolimus, prednisone
Albrecht [33]	PTCL-NOS	Autologous stem cell	3 months	“Good response”	Nodular skin lesions	Gemcitabine chemotherapy
Kajimoto [34]	PTCL-NOS (with pre-existing DLBCL)	Autologous HSCT	4 years	Not mentioned	Multiple lung nodules on CT	Not mentioned
Rajakarjar [19]	Hepato-splenic gamma delta type	Renal	7 years	DOD 2 weeks	POU and jaundice	Death before start of chemotherapy
Rajakarjar [19]	Hepato-splenic gamma delta type	Renal	8 years	DOD 4 months	AIHA	ROI
Draoua [35]	Hepato-splenic gamma delta type	Cardiac	9 years	DOD 9 months	N/A	ROI; CHOP x4 ESHAP; mitoxantrone/Navelbine/MTX/leukovorin
Lin [36]	ƴδ T-cell lymphoma	Kidney	5 years	Rapidly following Dx	Cough, sinus congestion, fever, night sweats	ROI, ganciclovir, 2-chlorodeoxyadenosine, G-CSF
Tey [37]	Hepato-splenic	Cardiac	15 years	Alive at 8-year f/u	Isolated neutropenia	Reduction of immunosuppression (ROI) HyperCVAD and MTX/HiDAC chemotherapy *
Elstrom [12]	Widespread T-cell PTLD	Renal Pancreas	2 years	Controlled at 23-month f/u	Abdominal pain, fever, weight loss, HA	ROI; CHOP; bexarotene
Haldas [38]	T-cell PTLD	Cardiac	8 years	Rapidly following Dx	Fever and chills	Death before start of Tx
Cardwell [39]	PTCL-NOS	Cardiac	21 months	Alive at 3 years, with relapse	Fevers and progressive dermatitis	ROI, chemo (unspecified), radiation, HSCT
Rajakarjar [19]	Extranodal NK	Renal	6 years	DOD 2 months	POU	Death before start of chemotherapy
Dalal [40]	T-cell lymphoma	Kidney	15 years	DOD 2 days after Dx	Thrombocytopenia and neutropenia	Comfort care
Draoua [35]	T-cell lymphoblastic	Cardiac	4 years	Alive at 48-month f/u	N/A	SWOG 9400 protocol
Kaminska [41]	T-cell lymphoma	Renal	4 years	DOD 14 months	Malaise, night sweats, fever, abdominal pain, weight loss	Switch to sirolimus and prednisone then CHOP
Kaminska [41]	T-cell lymphoma	Renal	4 years	Resolved in 12 months	Skin tumors	Switch to sirolimus and prednisone then surgery
Azhir [42]	Diffuse large T-cell lymphoma	Renal	4 years	Remission at 11-month f/u	Fever	ROI; gancyclovir; adriamycin, cyclophosphamide, vindesine, and prednisone.
Wang [43]	Diffuse large T-cell lymphoma	Bone marrow	2 years	Controlled 19 months after Dx	Bilateral cervical lymphadenopathy	All treatment stopped for personal reasons
Bregman [44]	Subcutaneous panniculitic-like T-cell lymphoma	Cardiac	3 years	DOD at 7.5 months	Nodular skin lesions	No change in Tx

* HyperCVAD consisted of cyclo-phosphamide 300 mg/m^2^ every 12 h for six doses (days 1–3), mesna 1800 mg/m^2^ over 3 days (days 1–3), vincristine 2 mg (days 4 and 11), doxorubicin 50 mg/m^2^ (day 4), and dexamethasone 40 mg/day (days 1–4 and 11–14). MTX/HiDAC cycle consisted of methotrexate 1 g/m^2^ over 24 h with leucovorin rescue, and Ara-C 3 g/m^2^ every 12 h for four doses (days 2–3). Both cycles included intrathecal methotrexate 12 mg on day 2 and Ara-C 100 mg on day 8. ** Chemotherapy regimen consisting of vincristine, doxorubicin (adriamycin), prednisolone, etoposide, cyclophosphamide, and bleomycin. **** Etoposide, prednisone, vincristine, and doxorubicin hydrochloride.

## Data Availability

No new data were created or analyzed in this study. Data sharing is not applicable to this article.
